# The association of aspirin use with severity of acute exacerbation of chronic obstructive pulmonary disease: a retrospective cohort study

**DOI:** 10.1038/s41533-018-0074-x

**Published:** 2018-02-21

**Authors:** Tadahiro Goto, Mohammad Kamal Faridi, Carlos A. Camargo, Kohei Hasegawa

**Affiliations:** Department of Emergency Medicine, Massachusetts General Hospital, 125 Nashua Street, Suite 920, Boston, MA 02114 USA

## Abstract

Little is known about the effect of long-term aspirin use on acute severity of COPD. We hypothesized that, in patients hospitalized for acute exacerbation of COPD (AECOPD), long-term aspirin use is associated with lower risks of disease severity (in-hospital death, mechanical ventilation use, and hospital length-of-stay). We conducted a retrospective cohort study using large population-based data from 2012 through 2013. Among 206,686 patients (aged ≥40 years) hospitalized for AECOPD, aspirin users had lower in-hospital mortality (1.0 vs. 1.4%; OR 0.60 [95% CI 0.50–0.72]; *P* < 0.001) and lower risk of invasive mechanical ventilation use (1.7 vs. 2.6%; OR 0.64 [95% CI 0.55–0.73]; *P* < 0.001) compared to non-users, while there was no significant difference in risks of non-invasive positive pressure ventilation use. Length-of-stay was shorter in aspirin users compared to non-users (*P* < 0.001). In sum, in patients with AECOPD, aspirin use was associated with lower rates of in-hospital mortality and invasive mechanical ventilation use, and shorter length-of-stay.

Increasing evidence suggests a potential benefit of antiplatelet therapy on chronic outcomes in patients with chronic obstructive pulmonary disease (COPD).^1,2^ For example, a recent analysis of an ongoing prospective cohort study revealed that antiplatelet therapy was associated with a lower 1-year mortality among individuals with COPD.^1^ However, little is known about the effect of aspirin use on acute outcomes (including mortality) in patients hospitalized for acute exacerbation of COPD (AECOPD).

To examine the association of aspirin use with severity of AECOPD, we conducted a retrospective cohort study using data from the State Inpatient Databases (SID) of seven US states (Arkansas, Florida, Iowa, Nebraska, New York, Utah, and Washington) from 2012 and 2013. Additional details of the SID may be found elsewhere.^3^ The institutional review board of Massachusetts General Hospital approved this study.

We identified all hospitalizations made by patients aged ≥40 years with a primary discharge diagnosis of COPD (*ICD-9-CM* codes: 491.21, 491.22, 491.8, 491.9, 492.8, 493.20, 493.21, 493.22, and 496), or those with a primary diagnosis of respiratory failure (codes: 518.81, 518.82, 518.84, and 799.1) and a secondary diagnosis of COPD.^4^ We included only the first hospitalization for AECOPD for each patient during the study period. Then, we further identified aspirin users by using the *ICD-9-CM* code for long-term (current) use of aspirin (V58.66) in any diagnostic field at the hospitalization.^5^

The outcome measures were in-hospital death (SID does not include the information on the cause of death), the use of mechanical ventilation (invasive mechanical ventilation [*ICD-9-CM* procedure code 96.04] and non-invasive positive pressure ventilation [NIPPV, code 96.30] assessed separately), and hospital length-of-stay (LOS). To examine the association between aspirin use and each outcome, we fit unadjusted and adjusted logistic regression or negative binomial regression models with generalized estimating equations to account for patient clustering within hospitals. We adjusted for age, sex, race/ethnicity, payer, median household income, 27 Elixhauser comorbidity measures (including heart failure, arrhythmias, and valvular diseases), hospitalization year, and hospital state. We repeated the analyses with (1) excluding patients who had any of the cardiovascular comorbidities that are commonly treated with aspirin (coronary artery diseases [codes, 410–414] and ischemic stroke [codes, 433–434]) and (2) excluding these potential aspirin users and patients with heart failure. All analyses were performed using STATA 14.1 (StataCorp, College Station, TX).

Overall, we identified 206,686 patients with hospitalization for AECOPD in the seven US states. Of these, 13,826 patients (7%) were aspirin users. Aspirin users were older, and more likely to have Medicare comorbidities, compared to non-users (all *P* < 0.001; Table 1). Aspirin users had a significantly lower in-hospital mortality compared to non-users (1.0 vs. 1.4%; adjusted OR 0.60 [95% CI 0.50–0.72] *P* < 0.001; Table 2). Additionally, aspirin users had a significantly lower rate of invasive mechanical ventilation use (1.7 vs. 2.6%; adjusted OR 0.64 [95% CI 0.55–0.73] *P* < 0.001), while there was no significant difference in the rate of NIPPV use (7.6 vs. 7.2%; adjusted OR 1.05 [95% CI 0.98–1.12] *P* = 0.20). Hospital LOS was significantly shorter in aspirin users compared to non-users (median, 3 days vs. 4 days), corresponding to −7% change in the adjusted model (95% CI −5 to −9%, *P* < 0.001). In the sensitivity analysis excluding the patients with history of coronary artery diseases, ischemic stroke, and/or heart failure, the point estimates of these associations did not change materially.

**Table 1:** Characteristics of patients hospitalized for acute exacerbation of chronic pulmonary obstructive disease, according to aspirin use

	Aspirin users	Non-users	
Characteristics	*n* = 13,826	*n* = 192,860	*P-*value
Age, year, median (interquartile range)	73 (65–81)	70 (59–79)	<0.001
Female sex	7229 (52)	112,549 (58)	<0.001
Race/ethnicity			<0.001
Non-Hispanic white	11,528 (86)	139,497 (75)	
Non-Hispanic black	867 (6)	19,134 (10)	
Hispanics	716 (5)	18,996 (10)	
Others	366 (3)	7250 (4)	
Payer			<0.001
Medicare	10,835 (78)	135,143 (70)	
Medicaid	902 (7)	21,012 (11)	
Private	1474 (11)	22,843 (12)	
Self-pay	238 (2)	7738 (4)	
Others	377 (3)	6116 (3)	
Quartiles for median household income			<0.001
1 (lowest)	3958 (29)	61,225 (33)	
2	4147 (31)	52,084 (28)	
3	3144 (23)	43,354 (23)	
4 (highest)	2333 (17)	30,680 (16)	
Number of comorbidities			<0.001
0–1	433 (3)	14,728 (8)	
2–3	4173 (30)	74,140 (38)	
≥4	9220 (67)	103,992 (54)	
Selected comorbidities			
Coronary artery diseases	6974 (50)	55,723 (29)	<0.001
Ischemic stroke	273 (3)	1821 (1)	<0.001
Hospital state			<0.001
Arkansas	1006 (7)	13,008 (7)	
Florida	6085 (44)	90,530 (47)	
Iowa	594 (4)	9431 (5)	
Nebraska	114 (1)	5512 (3)	
New York	3695 (27)	58,240 (30)	
Utah	76 (1)	2757 (1)	
Washington	2256 (16)	13,382 (7)	

*Note*: Data were shown as *n* (%) unless otherwise specified

**Table 2:** Unadjusted and adjusted associations between aspirin use and severity of acute exacerbation of chronic obstructive pulmonary disease

Outcomes	Aspirin users	Non-users	Unadjusted association (95% CI)	*P-*value	Adjusted association (95% CI)	*P-*value
Main analysis						
In-hospital death^a^	1.0% (0.8–1.1%)	1.4% (1.4–1.5%)	0.67 (0.56–0.80)	<0.001	0.60 (0.50–0.72)	<0.001
Invasive mechanical ventilation^a^	1.7% (1.5–1.9%)	2.6% (2.5–2.6%)	0.64 (0.56–0.74)	<0.001	0.64 (0.55–0.73)	<0.001
NIPPV use^a^	7.6% (7.2–8.1%)	7.2% (7.1–7.3%)	1.06 (0.99–1.13)	0.06	1.05 (0.98–1.12)	0.20
Hospital LOS, days, median (IQR)^b^	3 (2–5)	4 (2–6)	−6% (−4 to −7%)	<0.001	−7% (−5 to −9%)	<0.001
Sensitivity analysis 1^c^						
In-hospital death^a^	1.0% (0.8–1.3%)	1.3% (1.1–1.3%)	0.85 (0.67–1.09)	0.20	0.72 (0.56–0.93)	0.01
Invasive mechanical ventilation^a^	1.8% (1.5–2.1%)	2.4% (2.3–2.5%)	0.74 (0.61–0.89)	0.002	0.71 (0.59–0.87)	0.001
NIPPV use^a^	8.0% (7.3–8.7%)	7.1% (7.0–7.2%)	1.15 (1.05–1.27)	0.002	1.10 (0.99–1.21)	0.053
Hospital LOS, days, median (IQR)^b^	3 (2–5)	3 (2–5)	−2% (0 to −5%)	0.10	−5% (−2 to −8%)	<0.001
Sensitivity analysis 2^d^						
In-hospital death^a^	0.8% (0.6–1.1%)	1.0% (1.0–1.1%)	0.81 (0.59–1.11)	0.19	0.66 (0.47–0.92)	0.02
Invasive mechanical ventilation^a^	1.8% (1.5–2.2%)	2.4% (2.3–2.5%)	0.79 (0.64–0.97)	0.02	0.77 (0.62–0.95)	0.02
NIPPV use^a^	7.2% (6.5–7.9%)	6.2% (6.1–6.4%)	1.20 (1.08–1.34)	0.001	1.15 (1.03–1.29)	0.02
Hospital LOS, days, median (IQR)^b^	3 (2–5)	3 (2–5)	−1% (−4 to −2%)	0.52	−4% (−7 to −1%)	0.02

* CI* confidence interval, *NIPPV* non-invasive positive pressure ventilation, *IQR* interquartile range

^a^Odds ratio of aspirin use for each outcome in comparison with non-aspirin use, by using logistic regression model with generalized estimating equation to account for patient clustering within hospitals

^b^Percent change in hospital LOS, by using negative binomial model with generalized estimating equation to account for patient clustering within hospitals

^c^Among patients without coronary artery diseases or ischemic stroke, 6753 patients were aspirin users and 136,325 patients were non-users

^d^Among patients without coronary artery diseases, ischemic stroke, or heart failure, 5257 patients were aspirin users and 114,425 patients were non-users

Consistent with our findings, previous cohort studies have reported that, among patients with COPD, the use of antiplatelet therapy is associated with a lower long-term mortality.^6^ Additionally, an observational study of 1343 patients hospitalized for AECOPD in the UK reported that antiplatelet users (aspirin or clopidogrel) have a non-significantly lower odds of in-hospital mortality.^1^ Our population-based study from seven US states, with a much larger sample size, extends these findings by demonstrating the significant association of long-term aspirin use with lower acute severity and mortality of AECOPD.

The nature of aspirin-acute severity association in COPD warrants further investigation. The increased platelet activation in patients with AECOPD^7^ contributes to microvascular thrombosis that leads to organ ischemia and tissue damage.^8^ The use of aspirin may impede the inflammatory pathways secondary to the activated state of platelets and prevent microvascular thrombus formation by inhibiting the surface expression of adhesion molecules.^8^ These mechanisms for potential benefits of aspirin have also been indicated in other acute conditions accompanying systemic inflammation, such as sepsis and acute respiratory distress syndrome.^9^ Furthermore, the aspirin use potentially reduced the risk of cardiovascular diseases-related death, mechanical ventilation use, and longer LOS. This potential mechanism may be supported by the observed attenuated associations between aspirin use and severity of AECOPD in patients without cardiovascular diseases.

Our inferences may be limited by the low sensitivity of *ICD-9-CM* code for long-term (current) aspirin use.^6^ However, the results were consistent in the sensitivity analysis excluding potential aspirin users that addressed the under-coding among patients who should have been treated with antiplatelets. Additionally, our inferences might not be generalizable to patients with less-than-severe AECOPD. Nevertheless, our data remain highly relevant for 700,000 patients hospitalized yearly in the US,^10^ a population with high morbidity.

In sum, by using a large population-based data set, we found that long-term aspirin users have lower rates of in-hospital mortality and invasive mechanical ventilation use, and shorter LOS. Although causal inferences remain premature, in conjunction with the prior studies,^1,2,6^ aspirin—a widely used and inexpensive medication—may be a potential therapeutic option for patients with COPD.

## Data availability

The State Inpatient Databases can be purchased from the website of Healthcare Cost and Utilization Project (https://www.hcup-us.ahrq.gov).
